# Addressing anaemia in pregnancy in rural plains Nepal: A qualitative, formative study

**DOI:** 10.1111/mcn.13170

**Published:** 2021-07-09

**Authors:** Joanna Morrison, Romi Giri, Abriti Arjyal, Chandani Kharel, Helen Harris‐Fry, Philip James, Sushil Baral, Naomi Saville, Sara Hillman

**Affiliations:** ^1^ UCL Institute for Global Health University College London London UK; ^2^ HERD International Kathmandu Nepal; ^3^ Department of Population Health London School of Hygiene & Tropical Medicine London UK; ^4^ UCL Institute for Women's Health University College London London UK

**Keywords:** anaemia, gender, iron folic acid, participatory, pregnancy, qualitative, South Asia

## Abstract

Maternal anaemia prevalence in low‐income countries is unacceptably high. Our research explored the individual‐, family‐ and community‐level factors affecting antenatal care uptake, iron folic acid (IFA) intake and consumption of micronutrient‐rich diets among pregnant women in the plains of Nepal. We discuss how these findings informed the development of a home visit and community mobilisation intervention to reduce anaemia in pregnancy. We used a qualitative methodology informed by the socio‐ecological framework, conducting semi‐structured interviews with recently pregnant women and key informants, and focus group discussions with mothers‐in‐law and fathers. We found that harmful gender norms restricted women's access to nutrient‐rich food, restricted their mobility and access to antenatal care. These norms also restricted fathers' role to that of the provider, as opposed to the caregiver. Pregnant women, mothers‐in‐law and fathers lacked awareness about iron‐rich foods and how to manage the side effects of IFA. Fathers lacked trust in government health facilities affecting access to care and trust in the efficacy of IFA. Our research informed interventions by (1) informing the development of intervention tools and training; (2) informing the intervention focus to engaging mothers‐in‐law and men to enable behaviour change; and (3) demonstrating the need to work in synergy across individual, family and community levels to address power and positionality, gender norms, trust in health services and harmful norms. Participatory groups and home visits will enable the development and implementation of feasible and acceptable strategies to address family and contextual issues generating knowledge and an enabling environment for behaviour change.

**Key messages**

- Anaemia in pregnancy is one of the main causes of maternal deaths and adverse pregnancy outcomes in low‐ and middle‐income countries.
- We report findings from formative research to inform design of our interventional trial aimed at reducing anaemia in Nepal.
- Harmful gender norms restricted women's mobility, access to iron‐rich food and antenatal care. These norms also restricted fathers' roles. It is clear that improving knowledge alone will be insufficient to prevent anaemia.
- Interventions should work synergistically with families and communities to address harmful norms. Our research is an exemplar of how to incorporate context into the design of interventions.

## 1 INTRODUCTION

Anaemia in pregnancy affects 38% of women and is one of the main causes of maternal deaths and adverse pregnancy outcomes in low‐ and middle‐income countries (Daru et al., 2018; Rahman et al., 2016). Early identification and correction of anaemia in pregnancy are associated with improved maternal and neonatal outcomes (Stevens et al., 2013). Women in South Asia suffer disproportionately high rates of anaemia with 49% of pregnant women reported to be anaemic (Development Initiatives, 2020). In South Asia, early marriage and childbearing, high parity and short birth spacing, gender inequalities, lack of access to diverse and nutrient‐rich foods, infection and inadequate water, sanitation and hygiene facilities all contribute to high rates of anaemia (Chakrabarti et al., 2018). This interplay of factors indicates the need for complex interventions to tackle this problem (Balarajan et al., 2011).

Around half of anaemia in pregnancy is amenable to iron supplementation (World Health Organization [WHO], 2015). Preventive iron supplementation, often combined with iron folic acid (IFA), reduces the risk of maternal anaemia by 70% (Peña‐Rosas et al., 2015) and is recommended by the WHO (2016) as a routine component of antenatal care. However, its effectiveness depends on high compliance. Health systems factors and individual factors, such as side effects and lack of knowledge about the benefits of IFA, affect compliance (Galloway et al., 2002; Siekmans et al., 2018). Recent reviews have suggested that community‐based delivery mechanisms can improve access to IFA and enable delivery of context‐relevant antenatal counselling and nutrition programmes during pregnancy (Goudet et al., 2018; Kavle & Landry, 2018). Nutrition education and counselling interventions can reduce the risk of maternal anaemia by 30% (Webb‐Girard & Olude, 2012).

**FIGURE 1:**
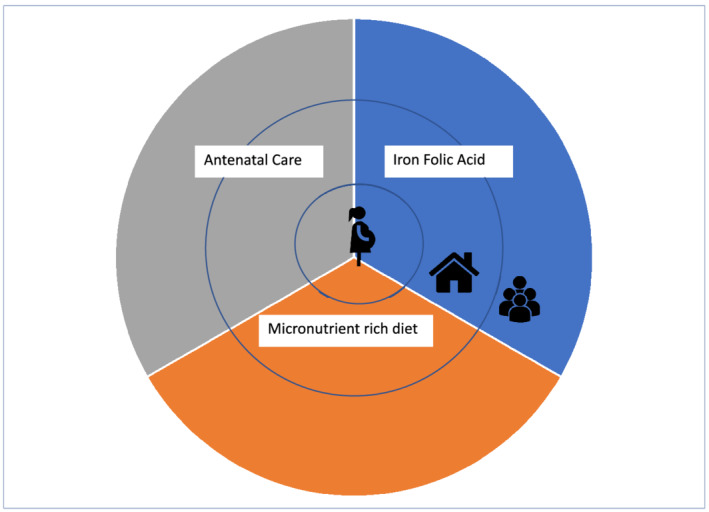
Conceptual framework: Target behaviours considered at individual, household and community levels

We report on findings from qualitative research in rural plains Nepal to explore the factors affecting anaemia in pregnancy. Based on the literature about drivers of anaemia in this context and the need to look beyond the individual when understanding behaviours, we developed a conceptual framework to enable analysis of the individual‐, family‐ and community‐level factors (and the interaction between them), which affect access to antenatal care, IFA adherence and consumption, and consumption of micronutrient‐rich foods. Intake of micronutrient‐rich foods and IFA are both direct determinants of anaemia (Peña‐Rosas et al., 2015) (Figure 1). Antenatal care is a distal determinant that provides evidence‐based interventions—early access to IFA and counselling around nutrition and care during pregnancy—which can help prevent and treat anaemia. Our conceptual framework is an adaption of Bronfenbrenner's (1977) ecological model, which is based on the premise that behaviour is affected by individual and environmental factors. These are defined as ecological systems that interact with each other and with the individual to influence behaviour.

It is clear that a complex intervention is necessary to address the causes of anaemia in pregnancy, and we will implement an intervention in 2021, which will contain home visits and community mobilisation. Each pregnant woman will receive two home visits, 4–6 weeks apart in mid‐pregnancy. Groups will be led through a participatory learning and action (PLA) cycle to identify issues of importance to them, to plan with communities how to address these issues and to implement and evaluate these plans over 14 months. This intervention is defined as a ‘collective’ group intervention as opposed to a ‘classroom’ or a ‘club’ (Gram et al., 2020), which engages participants in problem diagnosis and developing locally appropriate strategies to address the problems. The intervention, which builds on Freire's empowering education approach (Wallerstein, 1993), has proven effective in improving maternal and newborn survival in African and South Asian contexts (Prost et al., 2013) and addressing diabetes in Bangladesh (Fottrell et al., 2019), but there is only limited evidence of its effect on nutritional outcomes (Fottrell et al., 2018; Saville et al., 2018), and anaemia in pregnancy has never been measured as a trial outcome. In this paper, we focus on the individual and family factors that interact with community context to affect risk of anaemia in pregnancy and discuss how our findings informed the development of the intervention.

### 1.1 Anaemia in Nepal

Government efforts to improve nutrition in Nepal have intensified in recent years (Government of Nepal National Planning Commission, 2016). Yet despite these efforts, anaemia in pregnancy has shown little or no improvement.

The Demographic and Health Survey (DHS) shows that 41% of women aged 15–49 years and 46% of pregnant women were anaemic. Anaemia was most prevalent in the plains (52%), compared with the mountains (35%) or hills (29%) (Ministry of Health et al., 2017). Nutritional deficiencies and intestinal infections in Nepal contribute to the high prevalence of anaemia in women (Bondevik et al., 2000; Dreyfuss et al., 2000; Raut et al., 2016). The Nepal Micronutrient Status Survey found higher anaemia prevalence in women who had not consumed deworming tablets in the past 6 months compared with those who had (Ministry of Health and Population et al., 2018). Low dietary diversity among women results in inadequate consumption of iron‐rich foods and other micronutrients essential for healthy erythropoiesis and iron absorption (Gautam et al., 2019; Harris‐Fry et al., 2018; Henjum et al., 2014; Saville, Maharjan, et al., 2020). A recent analysis of DHS data found that women aged 20–29 years, with more than four children, who had experienced intimate partner violence and were from Provinces 1, 2 and 5 were at higher risk of anaemia (Rai et al., 2020). However, unexpectedly, anaemia was lower in the poorest quintiles and in overweight/obese women, and the concentration in prevalence has shifted from the poor to the better‐off between 2006/2011 and 2016 (Rai et al., 2020), thus demonstrating that more complex issues than access to food are at play.

### 1.2 Antenatal care in Nepal

Women receive free deworming medication and IFA tablets at antenatal care (60‐mg elemental iron and 400‐mcg folic acid) (Government of Nepal Ministry of Health and Population, 2064). Women are incentivised to have four antenatal care visits and are entitled to 800 NPR on completion of four visits in the fourth, sixth, eighth and ninth months of pregnancy (Government of Nepal, 2020). Antenatal care at the community level is provided by auxiliary nurse midwives and nurses (herein referred to as ‘nurses’) at Health Posts, Primary Health Centres and monthly community outreach clinics. Unpaid, locally resident Female Community Health Volunteers (FCHVS) also attend these clinics and can deliver IFA free of cost to women at home.

Antenatal care uptake has increased by 40 percentage points between 2006 and 2016, and in 2016, 84% of pregnant women had attended at least one check‐up. There are large urban–rural differences in access, number and timing of antenatal visits (Ministry of Health et al., 2017). Quantitative analysis has shown that access is related to education, socio‐economic status (Joshi et al., 2014) and caste and ethnicity (Pandey, 2013; Paudel et al., 2017), but qualitative research is needed to explore the reasons for low and delayed uptake in marginalised groups and engage them in design of interventions.

### 1.3 Gender in Nepal

Nepal is a patriarchal society, where boys and men are routinely privileged over women and girls. Traditionally, gender roles have varied somewhat with context, caste, ethnicity, religion and class, but feminist writing has demonstrated that dominant Hindu male state elites have sought to homogenise women and restrict them to the private, domestic sphere (Tamang, 2002). Women and girls are systematically disadvantaged by citizenship laws. For example, women who have been abandoned by husbands or widowed and women who have been raped or cannot name the father of their child are unable to pass citizenship, as a right, to their Nepali‐born children (Allison, 2017). They are also affected by practices that seek to control, seclude and restrict them such as the custom of expecting young married women to remain at home and not to be seen out in the community (Clark et al., 2019; Ghimire et al., 2013; Morrison, Basnet, et al., 2018). Limited decision‐making powers, early marriage, early pregnancy, overwork and neglect negatively affect women's health (Adaeze Nwokolo et al., 2020; Marphatia et al., 2020). Girls and women are responsible for housework, and their economic contribution to the family is largely unnoticed (Morrison, Dulal, et al., 2018). Men are expected to be family guardians and earn an income to support the family (Ghimire & Samuels, 2020). This, combined with factors such as dowry and patrilineal inheritance traditions, contributes towards norms of increased value of men over women. This often translates into men and children receiving more high‐status and luxury foods than women (Sudo et al., 2009). Even after accounting for the physiological differences in nutritional requirements, these intra‐household inequities persist (Harris‐Fry et al., 2018). Research shows that Nepalese households discriminate against pregnant women in the allocation of food, and they often receive smaller proportions of food compared with other family members (Gittelsohn, 1991; Harris‐Fry et al., 2018).

This literature on anaemia, antenatal care uptake and gender in Nepal indicates that the drivers of anaemia are complex and interacting and operate at multiple levels. Our research sought to explore the individual‐, family‐ and community‐level factors affecting antenatal care uptake, IFA intake and consumption of micronutrient‐rich diets among pregnant women in the plains of Nepal. Our findings were used to inform the development of an intervention to reduce anaemia in pregnancy, exemplifying how contextual understanding can be used to inform the design of appropriate interventions.

## 2 METHODS

### 2.1 Setting

Kapilvastu district, Province 5, is in the plains of Nepal and borders with Bihar, India. It has a population of ~572 000. Most families live in joint families with an average household size of 6.3 (Central Bureau of Statistics, 2012). The Human Development Index was 0.432 in 2014 (cf. 0.632 in Kathmandu) (Anand et al., 2014). Awadhi language is commonly spoken, and most of the population are Hindu (81%). The caste distribution comprises a majority of plains (Madhesi) groups, 13% disadvantaged low caste (Dalit), 17% indigenous (Janjati) and 18% Muslim (UNFCO, 2013). The district's literacy rate (55%) is lower than the national average (66%), and only 45% of women are literate (UNFCO, 2013). Labour migration to India is common, particularly among young men. The largest proportion of Nepalese who are classified as ‘absent’ comes from Province 5, and they make up 21% of Nepal's absent population (International Organization for Migration, 2019).

Province 5 has one of the lowest median ages of marriage for girls, at 18 years old (Ministry of Health et al., 2017), and reports indicate that child marriage is particularly prevalent among specific communities in Kapilvastu (Girls Not Brides et al., 2014). Patrilocal marriage practices whereby women move to their husband's home after marriage are usual, and patriarchal social norms that women should show respect through subservience to their husband and husband's family and should be under the protection and guardianship of that family are similar to those found in other plains districts of Nepal (Gram et al., 2018; Morrison, Dulal, et al., 2018). Women's mobility is strictly controlled in Kapilvastu, and a recent study showed that movement outside the house without permission was a common justification for intimate partner violence (Ghimire & Samuels, 2017). Women often move to their maternal home during pregnancy, and this is particularly common among Muslim women (Saville, Manandar, & Wells, 2020).

Province 5 has the second highest prevalence of anaemia in Nepal (44%) among women aged 15–49 years old (Ministry of Health Nepal et al., 2017); 67.3% of pregnant women in Province 5 attended four antenatal check‐ups, which is slightly higher than the national figure of 59% (Ministry of Health Nepal et al., 2017). Only 42% of women took the recommended dose of 180 IFA tablets, and 69% took deworming tablets during their pregnancy (Ministry of Health Nepal et al., 2017), indicating the need to improve these practices in order to improve anaemia. Women's consumption of iron‐rich foods is particularly low in the plains (Harris‐Fry et al., 2018), but iron adequacy in women's diets is low across Nepal (Saville, Maharjan, et al., 2020).

### 2.2 Sampling

We used our conceptual framework (Figure 1) to inform our sampling and our discussions with participants at the individual, family and community levels. We selected two rural and one urban municipalities where we could sample community members from marginalised (Muslim and Dalit) groups. Within these municipalities, we purposively sampled women and mothers‐in‐law whose child/grandchild was aged ≤6 months to explore their recent experience of a pregnancy and community norms. These women and mothers‐in‐law were from separate households. We sought to explore a breadth of experiences, so we sampled primiparous and multiparous women, women from marginalised and better‐off ethnic/caste groups and women from poor and better‐off households. It was challenging to find many fathers with young children, so we sampled fathers with a child aged <10 years to explore community norms and perceptions about fathers' role in pregnancy (Table 1). We sought triangulation of findings about community norms and an exploration of factors in the ‘community’ sphere of our conceptual framework through interviews with purposively sampled key informants and nurses from government health facilities. Key informants were identified after consulting with mothers‐in‐law, nurses and FCHVs and included non‐governmental organisation employees, FCHVs and religious leaders.

**TABLE 1:** Sample characteristics

	Women (16)	Fathers (20)	Mothers‐in‐law (19)
Number of children			
1	4	N/A	N/A
2 and above	12	N/A	N/A
Caste and ethnicity			
Plains low caste (Dalit)	6	8	11
Plains (Madhesi) marginalised	5	7	7
Plains indigenous (Janajati)	4		
Plains (Madhesi)	1	5	1
Religion			
Hindu	13	18	14
Muslim	3	2	5
Age of child/grandchild			
≤6 months	16	2	19
7 months to 10 years old	‐	18	‐
Estimated socio‐economic status			
Poor	4	N/A	N/A
Less poor	12	N/A	N/A

Abbreviation: N/A, not applicable.

### 2.3 Data collection

We recruited women, mothers‐in‐law and fathers through nurses and FCHVs. Researchers approached community members in their homes and nurses and key informants at their workplaces and took voluntary informed written or thumb‐print consent to participate. No one refused to participate, and data were collected locally in a place/time chosen by participants.

Between August and October 2019, we conducted 16 semi‐structured interviews (SSIs) with women, four SSIs with nurses, four key informant interviews, three focus group discussions (FGDs) with fathers and three FGDs with mothers‐in‐law (Table 2). Two trained Awadhi/Nepali‐speaking female researchers who lived in Kapilvastu collected data. Topic guides were developed in Nepali and Awadhi, which sought to explore our three key focus areas of IFA, micronutrient‐rich diet and antenatal care, as outlined in our conceptual framework, and definitions and experience of anaemia in this context. Researchers were mentored by RG and JM who observed five interviews and one FGD.

**TABLE 2:** Data collected in different respondent categories

Respondent type		Semi‐structured interviews	Key informant interviews	Focus group discussions, groups (total participants)
Mothers		16	‐	‐
Community key informants	Female community health volunteers	‐	1	‐
	Female supervisor of non‐governmental organisation nutrition programme	‐	1	‐
	Male religious leader	‐	1	‐
	Male community leader (as identified by community members)	‐	1	‐
Fathers		‐	‐	3 (20)
Mothers‐in‐law		‐	‐	3 (19)
Nurses		4	‐	‐
Total		20	4	6 (39)

### 2.4 Data management and analysis

**FIGURE 2:**
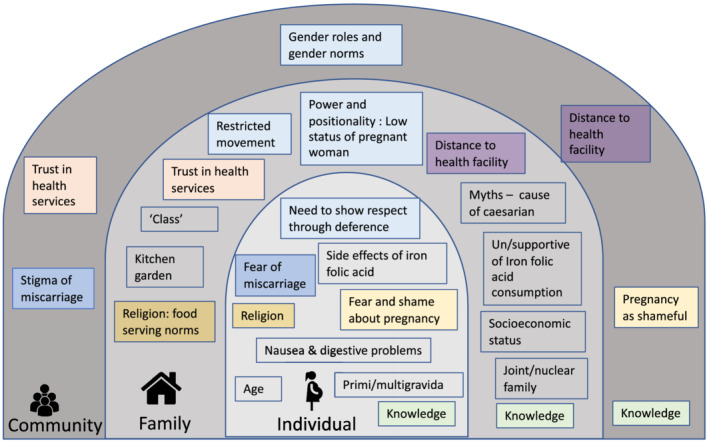
Factors affecting anaemia in pregnancy and their interactions at the community, family and individual levels. Legend: Colour‐matched boxes indicate related factors across community, family and individual levels

Data were digitally recorded, transcribed into Nepali and translated to English for analysis. For quality assurance, random sections of 10% of translations were checked against transcripts. We conducted descriptive content analysis, comparing data collected through different methods and from different respondent types. RG and JM read transcripts and made notes independently before discussing and agreeing on preliminary codes. Codes were developed inductively and deductively by RG and JM, and transcripts coded in Nvivo v11 qualitative analysis software. We used a socio‐ecological approach to analyse (1) individual‐level characteristics, knowledge and attitudes; (2) family‐level context including family relationships, norms and values; and (3) community‐level norms and environmental factors affecting maternal anaemia. This approach acknowledges that behaviours are affected by and affect multiple levels of influence and that the dynamic interplay between these levels is as important as the study of each level independently (Richard et al., 2011). This approach has been used in the analysis of maternal health care‐seeking behaviour (Kaiser et al., 2019; Shahabuddin et al., 2017) and in nutrition and anaemia research in low‐ and middle‐income countries (LMICs) (Gregson et al., 2001; Sedlander et al., 2020; Yilma et al., 2020). Figure 2 summarises our analysis at the three levels of influence. Narrative descriptions of codes were discussed with the wider team. We present our results pertaining to individual, family and community influences according to our conceptual framework (Figure 1), under headings of uptake of antenatal care, intake of micronutrient‐rich food and consumption of IFA, in order to develop recommendations to optimise these mechanisms.

### 2.5 Ethical considerations

The study was approved by the Nepal Health Research Council (353/2019) and the UCL Ethics Committee (14301/001) and London School of Hygiene and Tropical Medicine ethics committee (approval number: 16528).

## 3 RESULTS

### 3.1 Access to antenatal care

#### 3.1.1 Individual: Fear and shame

Accessing antenatal care according to protocol ensures early access to IFA and counselling around nutrition and care during pregnancy, which can help prevent anaemia. Most women accessed government health facilities for antenatal care to confirm their pregnancy, to check on their health and the health of their baby. A nurse felt that women were motivated by the opportunity to listen to the baby's heartbeat. They usually waited until the second or third month before attending antenatal care. Several young women were fearful about going outside while pregnant, and they felt vulnerable to evil spirits: ‘I was afraid in case my pregnancy would be affected by the Laghar [witchcraft]. I was also afraid of miscarriage’ (woman 110). Young and primigravida women often felt embarrassed about revealing their pregnancy: ‘Women don't go outside usually and if it is her first pregnancy then she doesn't want to come outside at all … she needs a guardian … some feel very shy’ (community leader NGO worker 101). Additionally, some felt that Muslim women were more likely to feel uncomfortable attending antenatal care: ‘Fewer Muslim women come for check‐ups because they have to remain under a scarf hiding their face’ (community leader, religious leader 103).

#### 3.1.2 Community: Shame and stigma

Mothers‐in‐law and community leaders suggested that young primigravida women were afraid of community gossip: ‘Everybody starts talking behind her back if she talks about her pregnancy … they are scared that they will be bad‐mouthed by everybody’ (mothers‐in‐law 101). It was unclear from the data whether women feared the stigma of miscarriage or being made to look foolish: ‘Community people make fun of you when you tell about it but later your menstruation reappears’ (mothers‐in‐law 102). Fathers were less understanding about women's shyness: ‘They do not talk about it with their in‐laws during their first pregnancy. They feel shy but they should not feel that way. It is a sign of weakness’ (fathers 104). Most women disclosed their pregnancy to their husbands first. Nurses said that women who were very poor or have many children do not usually attend the required four antenatal visits.

#### 3.1.3 Family: Power and positionality

Some women faced restrictions on their movement, particularly young or Muslim women, and women from conservative or ‘high‐class’ families: ‘My husband doesn't let me go outside that often’ (woman 108). Women accepted these restrictions: ‘I didn't go outside from my home because I am a daughter‐in‐law and it was my first baby. Daughters‐in‐law should not go outside’ (woman 103). The timing and frequency of antenatal care visits to health facilities were affected by her ability to arrange accompaniment. Her low status sometimes meant that fathers and mothers‐in‐law did not want to spend money and time taking pregnant women to the health facility, they did not feel antenatal care was beneficial and they expected their wives to deliver at home: ‘It requires money to take to the hospital. Why do we need to do the check‐up? Why should we take to the hospital if (the birth) can happen at home?’ (fathers 105). Mothers‐in‐law told us: ‘No one ever went for a check‐up from our home. Everybody had a healthy baby in the home. We vaccinated in the outreach clinic in the FCHVs house … the hospital is very far. How can we go that far? We don't go that far …. There is also no one at home to take daughters‐in‐law to the hospital … the ambulance takes Rs.300 for one‐way transport’ (mothers‐in‐law 106). Some nurses and community leaders felt that ‘(some families) give more importance to their work than going for check‐ups when it is time for crops to be planted or harvested’ (community leader 104). A woman in a nuclear family found it difficult to find time to go for antenatal care, given her lack of support with household chores.

When accessing antenatal care, pregnant women were accompanied by their mother‐in‐law, husband or sister‐in‐law, and a few went with the FCHV. Most fathers felt that women should have antenatal care, but this was discussed in a way that distanced them of responsibility to ensure access: ‘Some of them have an antenatal check‐up and some of them do not …. My wife never went. She did not have any complications during the delivery. This is how it works in a village’ (fathers 105). Women and mothers‐in‐law often reported that their husband/son directed the pregnant woman to attend antenatal care with a female family member: ‘When my daughter‐in‐law was pregnant, my son said: “Mother, she is not eating properly. Can you take her for a check‐up?” ’ (mothers‐in‐law 101).

Women's low status in the household made them fearful they would not be cared for sufficiently, which would affect their own and their baby's survival. Many women moved back to their maternal home during their pregnancy: ‘They go because they get more care in their maternal home than in their in‐laws' home …. They go to their maternal home thinking that their baby will have a better chance of survival’ (community leader 104). Nurses, women and community leaders reported that travel to the maternal home could disrupt the regularity of antenatal care.

#### 3.1.4 Community: Quality of antenatal care and trust

Most women felt well treated at antenatal clinics, and only a few women had had a bad experience. One woman was delayed by a sleeping nurse, and another woman was not examined as she had come before her scheduled appointment: ‘I felt sad when they told me to come back again without doing my check‐up even though I had pain in my stomach. They didn't do my check‐up even when I had come from so far away’ (woman 108). Distance to the health facility deterred some women, but many women used outreach clinics that were closer to home. The FCHV was not routinely consulted for antenatal care. An FCHV explained: ‘(FCHVs) don't know how to read and write. Since they are from the same village, people don't agree with them. If someone from outside talks to the community, people are more disciplined’ (community leader, FCHV 102). Fathers in all FGDs showed some distrust towards government health services. They complained about the cost of care and poor drug supply and discussed the need to use private health facilities: ‘The doctor brings the deworming tablets and vitamins, but they give them all to the people that they know personally. What can we do about this? How can we take (deworming tablets) if we do not get them at this health facility? We have to bring it from the private clinic’ (father 104). This distrust was not reported by other stakeholders.

### 3.2 Consumption of a micronutrient‐rich diet

#### 3.2.1 Individual: Nausea and digestive problems

Most women had suffered from digestive problems during pregnancy. Nausea, vomiting, diarrhoea, gas and bloating were common complaints. Pregnant women did not eat a lot of food because of these problems: ‘I did not like to eat, and my stomach felt heavy. I forced myself to eat. I only ate two times a day’ (woman 109).

#### 3.2.2 Individual and family: Knowledge

All types of respondents were aware that food consumption affected a woman's strength and the weight and appearance of her baby: ‘If she eats proper food, then she will get energy … if she has any problems with what she eats then it will be difficult to give birth’ (mothers‐in‐law 101). Green leafy vegetables, eggs, meat, fish, milk and fruit were thought to give strength and increase the amount of blood in the body. Beetroot and pomegranate were specifically mentioned. There were restrictions on eating pork among Muslims and buffalo among Hindus, and general consumption of meat was limited by its cost. Strict vegetarianism was uncommon.

#### 3.2.3 Family and community: Knowledge

Mothers‐in‐law and fathers felt that pregnant women should not eat stale food, and ‘dry food’ was considered insufficient in energy. Maintaining equilibrium in the body was discussed. Eating too much sour or spicy food, or food defined to have ‘heating’ properties, was not believed to be healthy. Participants of FGDs discussed their belief that too much food resulted in a difficult delivery or an expensive caesarean delivery, and nurses and community leaders corroborated the prevalence of these beliefs: ‘If (a woman) eats too much the baby gets too big inside their womb and they have to have a caesarean’ (father 104).

#### 3.2.4 Family: Power and positionality

In almost all families, fathers or mothers‐in‐law bought food and made decisions about what was cooked. ‘My husband goes to the market and brings food paid for by his labour work. How else could we eat? We eat because of that hard work’ (woman 102). Even in nuclear families, it was important to seek permission while purchasing for the household: ‘I take all the decisions because my husband is in India, but I have to ask him. I go to the market myself and I buy everything for the house, but I ask my husband and do everything’ (woman 110). It was easier to access vegetables if the family had a kitchen garden.

Restrictions on movement of younger women, lack of access to household money and cultural norms around respect and positionality in the household prevented them from making decisions or buying food. This was true across all types of households: ‘My mother‐in‐law makes the decision (about what to eat). I ate first during my pregnancy. I ate whenever I felt hungry. I ate everything—green leafy vegetables, fish, meat. My mother‐in‐law told me to eat whenever I liked’ (woman 108). Most women cooked and served food during their pregnancy, and therefore, they usually ate last to maintain ritual purity: ‘Women have to let others eat first then only eat later herself … the food remains pure if I let others eat first, and I can add food to their plate if they want’ (woman 101). Some women did not cook again when the food was finished by others. They ate salt, chilli and roti; rice and salt; or nothing at all. One woman said: ‘Who will cook again? My stomach is full if my family's stomach is full. I don't get hungry’ (woman 103).

Mothers‐in‐law and fathers acknowledged that pregnancy was a time of different nutritional need, and pregnant women should be given strength‐giving foods, and that they craved for sour and spicy food. Yet fathers felt financially unable to meet these needs: ‘(Pregnant women) have many desires, but they eat less food. We cannot provide so they eat simple food’ (father 106). Mothers‐in‐law and fathers expressed fatalism at not being able to provide: ‘God gives according to a person's faith’ (father 104). A few felt it was unrealistic to expect families to provide differentially even during pregnancy: ‘Where will we get money to buy food according to everyone's interest? We don't have money. (Pregnant women) eat whatever is cooked’ (community leader, FCHV). Some women, community leaders and nurses felt that families often did not prioritise the needs of pregnant women. One woman told us about her husbands' need to remain strong to provide for the family, which was more important than her need for a blood transfusion: ‘The doctor told me that I was anaemic. My husband was asked to provide blood. He is the one who earns money. If he gives me blood, then he will become weak’ (woman 104).

### 3.3 Consumption of IFA

#### 3.3.1 Individual: Knowledge

IFA was perceived to be good for pregnant women and their babies by most respondents, and most women took IFA during their last pregnancy: ‘It gives energy to the body, and you won't have weakness. The amount of blood in the body increases’ (woman 107). Almost all women reported learning about IFA from health workers, who they trusted, and they were grateful for their advice. Only one woman reported hearing that government IFA would be ineffective: ‘People had told me not to take it saying it won't do me good because it is a government medicine. They told me that it does nothing, but I didn't listen. I took it regularly and it did me good’ (woman 104). All fathers felt that most women stopped taking IFA as they ‘think that there is no benefit from taking IFA’ (father 106).

#### 3.3.2 Individual: Side effects

Five women suffered from side effects of IFA. Other respondents had heard about side effects from other women. Commonly perceived side effects were digestive problems, diarrhoea, vomiting, having black stool and dizziness. A few women said that the tablets had a bad smell. Those who continued taking IFA said that the side effects diminished over time. Only one woman stopped taking IFA because she felt unwell, but two community leaders and fathers in all FGDs thought this was more common: ‘Women get iron tablets, but they throw them away …. They do not like them … they feel like vomiting after having them’ (father 106). Nurses were unsure how many women regularly took IFA, because of side effects and the perception that they will make the baby large resulting in a costly and difficult delivery: ‘When we ask them why they haven't taken (IFA) they say that their child will get fatter if they take it which will lead to problems during delivery’ (nurse 101).

#### 3.3.3 Family: Power and positionality

Women reported that family members were usually supportive of them taking IFA. One mother‐in‐law brought IFA for her daughter‐in‐law, and one woman said that her family scolded her if she did not take IFA. Only one woman said that her sister‐in‐law would not bring IFA for her. Interestingly, some fathers felt that they were used as a scapegoat for women who did not want to take IFA: ‘(Women) say that their husband does not bring iron tablets for them while talking to others. They throw it away even if we bring it to them, then how would we know if they are taking iron tablets or not?’ (father 104). It is difficult to ascertain whether they were feeling defensive about their lack of knowledge about IFA consumption, or defending their refusal to resupply IFA because they thought it would be discarded anyway.

## 4 DISCUSSION

Our study revealed that the engagement of the family and community is crucial to addressing anaemia in pregnancy. Complex interventions are necessary to address the multiple causes of anaemia in this context. Pregnant women and other family members lacked awareness of iron‐rich food and how to manage side effects of iron supplementation, but it is clear that improving knowledge alone will be insufficient to prevent anaemia in pregnancy. Harmful gender norms restricted women's mobility, access to iron‐rich food and antenatal care. These norms also restricted fathers' roles. Few studies have explored the role of the family in Nepal to promote or enable behaviours that prevent or address anaemia in pregnancy. Families and communities maintain and reproduce harmful gender norms and interventions need to work synergistically with individuals, families and communities to address these norms and improve women's uptake of antenatal care, IFA intake and consumption of micronutrient‐rich food. We discuss our findings in the context of the literature and describe how the combined home visit and PLA community group intervention will engage with power and positionality, harmful gender norms, lack of knowledge about how to deal with side effects of IFA and lack of knowledge about iron‐rich food sources.

### 4.1 Engaging with power and positionality

Our research shows that women's position in the household is a major barrier to addressing anaemia in pregnancy. This positionality is driven by harmful gender norms and intergenerational hierarchies. In much of South Asia, women's unaccompanied mobility is restricted to protect her honour and the honour of her family (Bennett, 1983; Cameron, 1998). Her ability to arrange accompaniment to leave the household is related to her bargaining position and family ties within the household (Mumtaz & Salway, 2007; Simkhada et al., 2010). Women's seclusion within the household impedes their ability to build social support networks. Research has shown that women who have social capital may be more likely to access care and be cared for (Mumtaz & Salway, 2005) and may have better access to high‐quality food (Diamond‐Smith et al., 2020). We seek to build social networks and social capital through our community group‐based intervention, and we seek to make explicit how women's position within the home is related to anaemia in pregnancy through dialogue with families and community groups.

The intersection of gender with intergenerational hierarchies as well as class, caste and ethnicity also affect social capital, and this is important to consider in interventions (Harris‐Fry et al., 2017). For example, young women in high caste well‐off households may have access to iron‐rich food, but it might be unacceptable for them to leave the house. Home visits need to have a flexible format in order to respond to these differing situations.

### 4.2 Engaging with mothers‐in‐law

It is well known that the mother‐in‐law, or grandmother, is a key advisor in maternal health and nutrition in Nepal (Harris‐Fry et al., 2017, 2018; Masive, 2006; Morrison, Dulal, et al., 2018; Simkhada et al., 2010). Global reviews have shown mixed results of engagement of grandmothers in child health and development (Sadruddin et al., 2019; Tokhi et al., 2018), but this could be because presence is measured more often than their caregiving role (Chung et al., 2020). Research from South Asia has shown that living with a mother‐in‐law is associated with diminished woman's autonomy (Balk, 1994; Bloom et al., 2001; Jejeebhoy & Sathar, 2001) and increased risk of low body mass index (BMI) (Barker et al., 2006; Madjdian & Bras, 2016). Research from Malawi has shown that grandmothers may be more reluctant to dismiss traditionally held practices and adopt messages promoted by a peer‐led home education intervention (Scott et al., 2018). More research is needed on how to optimise the engagement of mothers‐in‐law for maternal and child health, considering their household position in this patriarchal society where they may be forced to prioritise the needs of earners and those more powerful in the household as opposed to pregnant women (Gram et al., 2018). We see the grandmother as a key stakeholder in the intervention, and we will build on their role as advisors and caregivers (Aubel, 2012; Tomlinson et al., 2014), encouraging them to play an active role in preventing anaemia in pregnancy during home visits and participating in community groups. Through their participation in community groups and home visits, they can help to develop an enabling environment to address the causes of anaemia.

### 4.3 Engaging men

Our data show men to be disengaged in maternal health, and although this may have been amplified by our focus group method—whereby participants might have felt pressure to express gender normative values while among peers—our findings were triangulated by other data sources. Established patriarchal gender norms of men as economic providers and decision‐makers, and women as dependents and homemakers, place maternal health within the female domain. Women's deference to the authority of her husband and to her mother‐in‐law is an expression of femininity, appropriate behaviour and respect (Gram et al., 2018; Morrison, Dulal, et al., 2018). This can prevent women from speaking up for what they need or want and can restrict men from playing a more active role in the care of pregnant women (Mullany, 2006). Men can be stigmatised in this context if they participate in housework or speak up for gender equality and they feel a burden to fulfil their role as provider (Ghimire & Samuels, 2017). Interventions seeking to build on the provider role of men in South Asia have had some success (Mullany et al., 2007; Nguyen et al., 2018), but the global evidence is less clear. Reviews of the effectiveness of engaging fathers to improve maternal and child health are mixed (Tokhi et al., 2018), partly because the pathways of influence are often inadequately articulated (Yargawa & Leonardi‐Bee, 2015), and coverage and engagement with interventions are often not reported (Martin et al., 2020). Gender‐normative perspectives held by health providers as well as families often hinder the engagement of men beyond their existing roles, and studies have suggested that facilitating joint decision‐making in couples is more beneficial than a separate focus (Mullany et al., 2005; Panter‐Brick et al., 2014). We will engage with men in three ways: (1) as family members through encouraging them to play an active role in preventing anaemia in pregnancy during home visits; (2) supporting community groups to discuss how they would like to engage with men; and (3) through inviting them to large community meetings where groups plan community actions. They may also play an important role in implementing community actions. Engagement with men will be led by families, groups and communities enabling them to address patriarchal norms in ways that they feel comfortable.

### 4.4 Engaging with FCHVs

FCHVs are tasked with running one women's group per month to raise awareness about health issues, and they are also expected to make home visits providing IFA and contraception, free of cost. They are an important link between the community and the health facility, but they are an unpaid and largely unsupervised cadre who are overburdened with many community health promotion campaigns (Khatri et al., 2017; Schwarz et al., 2014). A national survey in 2015 found that 54% were illiterate (Advancing Partners and Communities, 2015). Research has shown that they can find participatory discussion difficult to implement, being more used to providing services and giving health education (Morrison et al., 2020). Other studies have shown that they work best if they are supported and supervised (Neupane et al., 2018; Saville et al., 2018). Therefore, we will recruit and train a female nutrition assistant (NA), who is educated to at least Class 5, to co‐ordinate with FCHVs to arrange home visits of pregnant women. At the community level, NAs will convene community groups with the FCHV to identify the causes of anaemia at the family, community and health systems levels and plan, implement and evaluate actions to address anaemia with communities through a PLA cycle.

### 4.5 Developing knowledge and enabling action through a dialogical approach

We identified several areas where increased information could help households and communities address anaemia. For example, beetroot and pomegranate were specifically mentioned as iron‐rich foods (which is an erroneous perception), and eating dry food was discouraged. It would be beneficial for families to understand that eating dry food may help with nausea and for families to learn to identify iron‐rich locally available foods. However, we also found large variance in women's experiences of taking IFA and family members' perceptions of the benefits and quality of both IFA and antenatal care. Given this heterogeneity of experiences and the community‐level constraints to changing behaviour, simply giving information will not be sufficient to reduce anaemia. We seek to build capacity for households and communities to take action on the basis of knowledge and create an enabling environment for behaviour change. We will use tan empowering education approach informed by Paulo Freire's theory of education (Wallerstein, 1993).

NAs will initiate dialogue in groups and during home visits using a ‘trigger’ (Wallerstein & Bernstein, 1988). A trigger is a representation of an identified issue to help participants discuss an issue. This can take different forms. During home visits, our triggers will be stories based on information from our formative research about barriers to addressing anaemia. Participatory groups will use a different type of trigger each month using information from our formative research to trigger dialogue about different topics. The NA will ask the group/family questions about the story/game/quiz asking them to consider how the issues identified apply to their community and their situation. The NA and the group/family will learn together, collectively identifying their own problems, and begin to analyse the conditions that contribute to those problems. Through this critical reflection, they will be supported to develop strategies that are appropriate for the problem and the context. They can also develop strategies that they feel comfortable with (Wallerstein, 1993). An example from the home visit is presented in Table 3. Groups will follow this process over a 9‐month period, with discussions focusing on anaemia in pregnancy; antenatal check‐ups and IFA; IFA and side effects; micronutrient‐rich food and ways to increase absorption of iron; identifying support persons; and gender roles and how to engage with men. After nine meetings, groups will then organise larger community meetings whereby they will share the issues and suggested strategies with community members and lead on the planning and implementation of these over the next 5 months. We will provide NAs with a reference manual, which has been developed on the basis of our formative research. This manual uses formative data (not presented here) about locally available sources of nutrient‐rich food, locally appropriate methods of increasing absorption of iron in food and locally appropriate ways to reduce nausea and side effects of IFA. We will also give NAs a home visit discussion manual, a PLA group manual, comprehensive training on facilitation skills and field‐level supervision. We will use vignettes and role play in our training to ensure that NAs are prepared for a variety of community/family situations and that they are able to facilitate discussions as opposed to educating groups/families.

**TABLE 3:** Example of a home visit discussion

Issues identified by the family	Follow‐up questions	Advice	Action to resolve issue
Fear/embarrassment	What are the reasons that PW feels fearful/embarrassed? What would help PW feel better about ANC? What would you like to do about this?	‐ You can ask someone to be with PW at ANC. ‐ It's okay to go for an ANC visit at any time—even if PW attends after several months, or she has missed visits. ‐ The health worker will find out the position of foetus and listen to the heartbeat of the foetus. They can tell you (PW and family) if everything is ok. ‐ PW can visit her nearest outreach clinic for basic ANC. ‐ PW can have ANC at any public health facility.	What would you like to do about this? What would make it possible to this? What support would you need to do this? ‐ What action could you take to make this possible?

Abbreviations: ANC, antenatal care; PW, pregnant woman.

### 4.6 Limitations

Our maximum variation sampling made it more difficult to build knowledge about the intersections between intergenerational hierarchies as well as class, caste and ethnicity, but further exploration throughout the intervention will be possible through participatory discussion. The roles of family members such as grandfathers, sisters‐in‐law, maternal grandmothers or other family members from the maternal home were not explicitly explored in this study, but these stakeholders will be engaged in the home visits and PLA intervention.

## 5 CONCLUSION

Anaemia in pregnancy is a persistent problem in South Asia, and a contextual understanding of the causes of anaemia is necessary to develop responsive interventions. Our formative qualitative research is an exemplar of how to incorporate context into the design of interventions. We have revealed the complexity of factors driving anaemia and the cross‐cutting role of the family in addressing these drivers. Gender norms that place maternal health in the female domain, where men are not expected to participate beyond their provider role, are harmful and limit the agency of men and women to address anaemia in pregnancy. Our intervention will use a problem‐posing dialogical methodology, drawing on Freire's empowering education approach (Wallerstein, 1993), to enable families and communities to identify problems, to critically analyse the drivers of those problems and to develop and implement actions to change their situation.

## CONFLICTS OF INTEREST

The authors declare that they have no conflicts of interest.

## CONTRIBUTIONS

JM, AA and PJ designed the study. JM, RG and AA supervised and trained researchers, analysed the data and drafted recommendations. NS, HHF, PJ, SH and CK commented on analysis and development of recommendations. SB and SH provided management oversight. JM and RG wrote the paper. All authors have read and approved the final manuscript.

## Data Availability

The data that support the findings of this study are available from the corresponding author upon reasonable request.
